# Carbohydrate antigen 125 and carcinoembryonic antigen in the differentiation of tuberculous peritonitis and peritonitis carcinomatosa

**DOI:** 10.18632/oncotarget.17355

**Published:** 2017-04-21

**Authors:** Huan Tong, Yang Tai, Cheng Ye, Hao Wu, Lin-Hao Zhang, Jin-Hang Gao, Zhao-Ping Yan, Zhi-Yin Huang, Cheng-Wei Tang

**Affiliations:** ^1^ Department of Gastroenterology, West China Hospital, Sichuan University, Chengdu, PR China; ^2^ Division of Peptides Related with Human Diseases, State Key Laboratory of Biotherapy, West China Hospital, Sichuan University, Chengdu, PR China

**Keywords:** tuberculosis peritonitis, peritonitis carcinomatosa, tumor markers, carbohydrate antigen 125, carcinoembryonic antigen

## Abstract

Tumor markers could increase in both tuberculous peritonitis and peritonitis carcinomatosa, confusing the differentiation of these diseases. This study aimed to better understand the extent of elevation and diagnostic efficacies of carbohydrate antigen 125 (CA 125), carcinoembryonic antigen (CEA) and combinative use of them in tuberculous peritonitis and peritonitis carcinomatosa. Of 2998 patients reviewed, 101, 120 and 71 patients were assigned to TBP group (tuberculous peritonitis), non-OCA group (non-ovarian carcinoma-related peritonitis carcinomatosa) and OCA group (ovarian carcinoma-related peritonitis carcinomatosa), respectively. The composite index was calculated by CA 125 multiplying CEA. Receiver operator characteristic curves for CA 125, CEA and composite index were acquired. As a result, CA 125 value in OCA group was higher than other two groups (serum CA 125: *P* < 0.001; ascites CA 125: *P* < 0.001). On the other hand, non-OCA group had the highest CEA value among three groups (serum CEA: *P* < 0.001; ascites CEA: *P* < 0.001). Area under curves of serum/ascites composite index and serum/ascites CEA were larger than those of serum/ascites CA 125. Furthermore, ascites and serum composite index displayed the best sensitivity (0.907) and specificity (0.989), respectively. In conclusion, CA 125 increases in tuberculous peritonitis and non-ovarian carcinoma-related peritonitis carcinomatosa, but it elevates more in ovarian carcinoma-related peritonitis carcinomatosa. CEA is found to increase more significantly in non-ovarian carcinoma-related peritonitis carcinomatosa. CEA and composite index are helpful in distinguishing peritonitis carcinomatosa from tuberculous peritonitis, but composite index is slightly superior to CEA in the differential diagnosis.

## INTRODUCTION

Tuberculous peritonitis (TBP) and peritonitis carcinomatosa (PC) are the main causes of exudative ascites. It is important to distinguish TBP from PC for their different treatment strategies and prognoses. However, the differentiation is not an easy task because both of them may present similar symptoms such as abdominal tenderness [1]. Bacteria culture and interferon-γ release assays (IGRAs) are not able to solve the problem completely since bacteria culture is time-consuming with limited sensitivity of less than 80% [2], and the long waiting period for the result might compromise the prognosis of tuberculosis [3]. Moreover, IGRAs require high cost and complicated technology [4–6], making it unavailable in remote areas. Additionally, as IGRAs were mainly applied to pulmonary tuberculosis and high risk group [7–10], it is still uncertain whether IGRAs are conferred to enough sensitivity and specificity in extrapulmonary tuberculosis [11].

Tumor markers were developed to distinguish malignancy from benign conditions, but it also turns out confusing under certain circumstances. Carbohydrate antigen 125 (CA 125), usually elevated in ovarian neoplasms, has also been reported to elevate in TBP by many papers [12–20]. However, they have not properly determined the disparity of CA125 levels in TBP and PC. Moreover, carcinoembryonic antigen (CEA), which is mainly found to elevate significantly in adenocarcinoma involving abdominal organs [21], were seldom investigated in TBP.

Therefore, this study aimed to determine the levels of CA 125 and CEA, and the diagnostic efficacy of CA 125 and/or CEA to differentiate TBP from PC in ascitic patients with TBP or PC.

## MATERIALS AND METHODS

### Patients

Patients with ascites diagnosed with TBP or PC in West China Hospital between 2009 and 2013 were reviewed. TBP was confirmed by either positive pathological findings (acid-fasting stain/smear positive and/or caseous necrosis), or strong clinical evidence consistent with TBP and responsive to the full course of tuberculosis chemotherapy. Meanwhile, PC was diagnosed by findings of carcinoma cells in ascites and/or positive biopsy findings.

Patients were excluded if any item listed below was fulfilled: 1) neither CA 125 nor CEA was measured; 2) patients with TBP suffered from tuberculosis in other sites (eg. lung, pleural cavity and bone); 3) treatment-experienced to tuberculosis or tumor; 4) comorbidity of liver cirrhosis, congestive heart failure or nephrotic syndrome; 5) high-dosage intake of diuretics, or large volume paracentesis before measurements of CA 125 and CEA.

### Study design

The patients with TBP were assigned to TBP group. As the traditional opinion held that CA125 elevated significantly in female patients with the ovarian tumor, the patients with PC were sequentially classified into two groups by whether the primary tumor location was ovarian or not: the ovarian carcinoma-related PC group (OCA group) and the non-ovarian carcinoma-related PC group (non-OCA group).

The baseline characteristics of each patient were collected. Both CA 125 and CEA were measured by electrochemiluminescence immunoassay with the same kit (Roche, Basel, Switzerland), and their levels in serum and ascites were recorded respectively. Meanwhile, data incompleteness for CA 125 and CEA was also recorded for the bias evaluation. The relationships between tumor markers and four factors (age, gender, underlying condition and ascites volume) were also detected to evaluate the influence of potential influential factors on tumor markers.

In the previous clinical work, we found a trend in patients that TBP and non-OCA PC had lower levels of CA 125 than that of OCA-related PC, and non-OCA-related PC and OCA-related PC had higher levels of CEA than that of TBP. Therefore, we proposed a hypothesis that TBP and PC might be differentiated by CEA multiplying CA 125 for the combinative use of CA 125 and CEA might magnify the power of CA 125 to differentiate TBP from PC. Therefore, the composite index (CI) was calculated by CA 125×CEA in serum and ascites separately, and receiver operator characteristics (ROC) curves and area under curves (AUCs) for CA 125, CEA and CI were also obtained.

### Statistical analysis

Data were mainly tested by Chi-square test or analysis of variance (ANOVA) depending on the type of data, and the nonparametric test (Wilcoxon rank sum test or Nemenyi test) was employed if the quantitative data lacked the homogeneity of variances. Pearson correlation analysis was applied to determine the correlation between the serum level and the ascites level of tumor markers. Additionally, the ROC curve was applied to determine the power of different parameters (serum and ascites CA 125, CEA and CI) to distinguish PC from TBP. All the statistical work was conducted with SPSS 19.0 software.

## RESULTS

### Patient enrollment and data incompleteness

**Figure 1:**
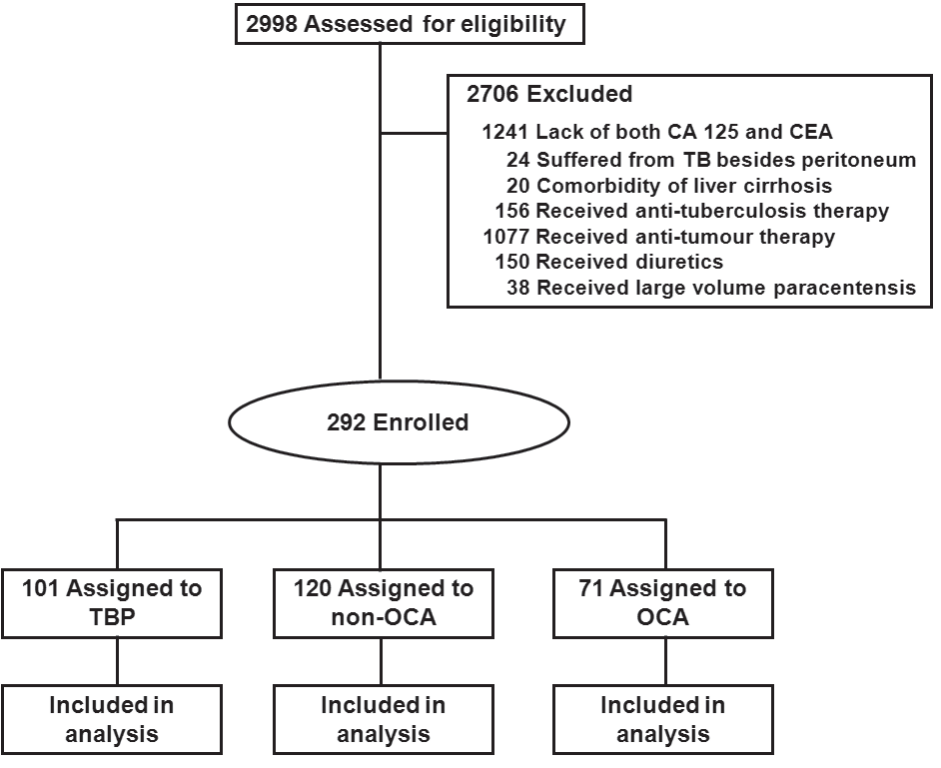
Flow of patients Abbreviations: CA 125, carbohydrate antigen 125; CEA, carcinoembryonic antigen; non-OCA: non-ovarian carcinoma; OCA: ovarian carcinoma; TB: tuberculosis; TBP, tuberculous peritonitis.

During January 2009 to December 2013, 2998 patients with ascites were diagnosed with TBP or PC in West China Hospital. After initial screening, 2706 patients were excluded. Finally, 101, 120 and 71 patients met our criteria and were assigned to TBP group, non-OCA group and OCA group, respectively (Figure 1). As for data incompleteness, it was generally comparable except for serum CA 125 (Table 1).

**Table 1:** Basic characteristics and data incompleteness

	TBP	non-OCA	OCA	*P*
**N**	101	120	71	NA
**Demography**				
Age (years, mean ± SD)	40.8 ± 18.4†‡	61.9 ± 12.8†	56.3 ± 12.4	< 0.001
Gender (male/female, *n*)	45/56*	71/49*	0/71	< 0.001
**Symptoms and signs**				
Abdominal pain, *n* (%)	63 (62.4)	78 (65)	38 (53.5)	0.279
Abdominal tenderness, *n* (%)	79 (78.2)	90 (75)	46 (64.8)	0.131
Constipation, *n* (%)	7 (7.9)	9 (7.5)	5 (7.0)	0.985
Diarrhoea, *n* (%)	24 (23.8)*	19 (15.8)	5 (7.0)	0.014
Fever, *n* (%)	44 (43.6) *#	8 (6.7)	7 (9.9)	< 0.001
Night sweat, *n* (%)	29 (28.7) *#	3 (2.5)	5 (7.0)	< 0.001
**Grading of ascites**				
Grade 1, *n* (%)	0 (0)	0 (0)	0 (0)	0.500
Grade 2, *n* (%)	64 (63.4)	84 (70.0)	45 (63.4)	
Grade 3, *n* (%)	37 (36.6)	36 (30.0)	26 (36.6)	
**Data incompleteness**				
Serum				
CA125 missing, *n* (%)	8 (7.9)	7 (5.8)	4 (5.6)	0.775
CEA missing, *n* (%)	9 (8.9)	19 (15.8)	13 (18.3)	0.166
Ascites				
CA125 missing, *n* (%)	52# (51.5)	82 (68.3)	36 (50.7)#	0.014
CEA missing, *n* (%)	63 (62.4)	90 (75)	44 (62.0)	0.072

*P* < 0.05: † *vs*. OCA, ‡ *vs*. non-OCA; *P* < 0.0125: * *vs*. OCA, # *vs*. non-OCA.

*P* values derived via ANOVA or chi-square test as appropriate.

The grading of ascites refers to EASL clinical practice guidelines on the management of ascites, spontaneous bacterial peritonitis, and hepatorenal syndrome in cirrhosis, which was issued in 2010. Detailedly, grade 1 ascites is defined as mild ascites only detectable by ultrasound; grade 2 ascites is defined as moderate ascites evident by moderate symmetrical distension of abdomen; grade 3 ascites is defined as large or gross ascites with marked abdominal distension.

Abbreviations: ANOVA, analysis of variance; CA 125, carbohydrate antigen 125; CEA, carcinoembryonic antigen; N, number; non-OCA, non-ovarian carcinoma; OCA, ovarian carcinoma; SD, standard deviation; TBP, tuberculous peritonitis.

### Baseline characteristics

The patients in TBP group had a smaller average age than non-OCA group and OCA group, and the gender ratios of three groups were also different from each other (Table 1). For symptoms and signs, TBP group had larger proportions of manifestations such as diarrhea, fever and night sweat (Table 1). In aspect of the grading of ascites, the majority of patients in each group were categorized to grade 2 (Table 1).

The diagnoses of 79 patients (81.8%) in TBP group were confirmed by pathology and the remains (22 patients, 18.2%) were established if they were responsive to the empirical full course of tuberculosis chemotherapy. In non-OCA group, primary tumor foci were located in stomach (34 patients, 28.3%), followed by unidentified sites (29 patients, 24.2%), pancreas (23 patients, 19.2%), colon (16 patients, 13.3%), biliary tree (14 patients, 11.7%) and rectum (4 patients, 3.3%).

### CA 125 and CEA levels in serum and ascites

The serum and ascites CA 125 levels in TBP group and non-OCA group were both lower than those in OCA group. Differently, as for the serum and ascites CEA, non-OCA group took the highest rank, followed by OCA group and TBP group (Table 2). The correlation of the tumor marker level in serum and ascites existed, no matter whether it was CA 125 or CEA (CA 125: r^2^ = 0.641, P < 0.001; CEA: r^2^ = 0.536, P < 0.001).

**Table 2:** Levels of CA 125 and CEA in serum and ascites

				TBP	non-OCA	OCA	*P*
Serum	CA125 (U/ml)		*N*	93	113	67	NA
		Overall patients		344.5 (160.5-563.6)*	227.4 (101.3-475.7)*	1219.0 (312.5-2781.0)	< 0.001
			*N*	39	65	0	NA
		Male		279.2 (156.3-498.0)	237.7 (94.6-442.6)	NA	0.199
			*N*	54	48	67	NA
		Female		392.5 (178.4-614.0)*	227.0 (108.5-511.9)*	1219.0 (312.5-2781.0)	< 0.001
	CEA (ng/ml)		*N*	92	101	58	NA
		Overall patients		1.2 (0.8-2.2)*#	148.1 (16.1-1000.0)	8.8 (5.4-26.4)#	< 0.001
			*N*	41	60	0	NA
		Male		1.2 (0.7-2.2)†	197.4 (21.1-1000.0)	NA	< 0.001
			*N*	51	41	58	NA
		Female		1.4 (0.8-2.2)*#	39.5 (13.8-906.4)	8.8 (5.4-26.4)	< 0.001
Ascites	CA125 (U/ml)		*N*	49	38	35	NA
		Overall patients		746.3 (337.6-1232.0)*	984.5 (584.6-1618.3)*	4454.0 (1404.0-5000.0)	< 0.001
			*N*	24	19	0	NA
		Male		638.8 (218.8-1238.5)†	936.9 (855.2-1377.0)	NA	0.045
			*N*	25	19	35	NA
		Female		804.7 (520.0-1206.0)*	1032.0 (469.9-1921.0)*	4454.0 (1404.0-5000.0)	< 0.001
	CEA (ng/ml)		*N*	38	30	27	NA
		Overall patients		0.7 (0.4-1.5)*#	413.9 (14.3-1000.0)	8.1 (3.9-260.3)	< 0.001
			*N*	17	16	0	NA
		Male		0.8 (0.5-2.1)†	413.9 (61.5-990.8)	NA	< 0.001
			*N*	21	14	27	NA
		Female		0.6 (0.4-1.3)*#	309.5 (4.6-1000.0)	8.1 (3.9-260.3)	< 0.001

*P* values derived via Nemenyi test. *P* < 0.05: † *vs*. non-OCA; *P* < 0.0125: * *vs*. OCA, # *vs*. non-OCA. All data in this part are presented as median (25^th^ percentile - 75^th^ percentile).

Abbreviations: CA 125, carbohydrate antigen 125; CEA, carcinoembryonic antigen; N, number; NA, not applicable; non-OCA, non-ovarian carcinoma-related peritonitis carcinomatosa; OCA, ovarian carcinoma-related peritonitis carcinomatosa; TBP, tuberculous peritonitis.

Moreover, the female had higher levels of serum and ascites CA 125 than the male. Patients with the age ≥ 45 years bore higher levels of serum CEA, ascites CA 125 and ascites CEA, compared with those with the age < 45 years. However, the grading of ascites did not influence the levels of serum/ascites CA 125 or serum/ascites CEA (Table 3).

**Table 3:** Influence of gender, age and ascites volume on CA 125 and CEA

			Gender			Age			Grading of ascites		
			Male	Female	*P*	< 45 yrs	≥ 45 yrs	*P*	Moderate	Severe	*P*
Serum	CA 125 (U/ml)	N	104	169		78	195		181	92	
			260.1 (107.9-466.0)	453.9 (184.7-1299.5)	< 0.001	323.9 (136.0-557.8)	380.4 (135.9-930.0)	0.157	296.9 (128.7-741.6)	441.6 (182.6-898.2)	0.152
	CEA (ng/ml)	N	101	150		76	175		164	87	
			13.0 (1.3-309.3)	6.0 (1.3-32.0)	0.084	1.3 (0.8-3.0)	13.2 (2.8-229.0)	< 0.001	6.7 (1.2-82.0)	7.0 (1.8-95.7)	0.422
Ascites	CA 125 (U/ml)	N	43	79		36	86		78	44	
			903.6 (352.9-1281.0)	1290.0 (632.4-5000.0)	0.001	893.4 (367.4-1215.0)	1327.5 (714.5-4283.0)	0.001	1007.2 (538.0-3187.0)	1178.5 (743.0-1905.3)	0.485
	CEA (ng/ml)	N	33	62		27	68		59	36	
			2.3 (0.8-413.9)	4.3 (0.6-99.4)	0.760	0.9 (0.4-26.2)	6.3 (1.0-397.9)	0.010	7.3 (0.6-794.1)	2.4 (0.8-29.9)	0.433

All data in Table 3 are presented as median (25^th^ percentile - 75^th^ percentile).

*P* values derived via Wilcoxon rank sum test.

Abbreviations: CA 125, carbohydrate antigen 125; CEA, carcinoembryonic antigen; N, number.

Considering all patients in OCA group were female, the results above of the influence of gender on the tumor makers might include significant bias. Therefore, when OCA group was excluded, and only TBP group and non-OCA group were compared, it turned out that no differences were found in serum CA 125, ascites CA 125 or ascites CEA (P for serum CA 125 0.126, serum CEA 0.015, ascites CA 125 0.519 and ascites CEA 0.132).

### ROC curves to differentiate PC from TBP

**Figure 2:**
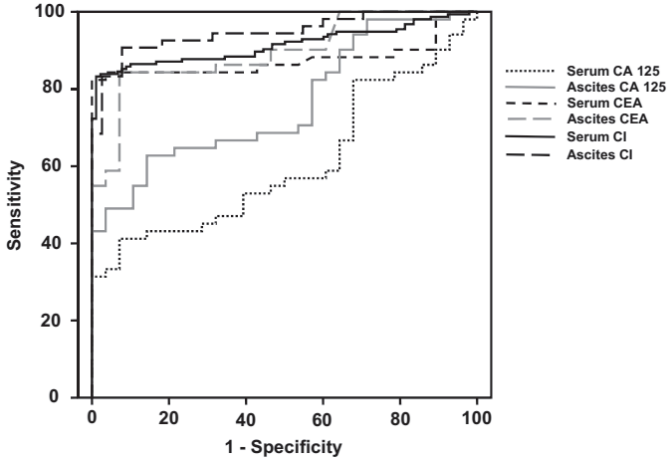
ROC curves of CA 125, CEA and CI in distinguishing PC from TBP Abbreviations: CA 125, carbohydrate antigen 125; CEA, carcinoembryonic antigen; CI, composite index; PC, peritonitis carcinomatosa; ROC, receiver operator characteristic; TBP, tuberculous peritonitis.

Among the different ROC curves, serum CI, ascites CI, serum CEA and ascites CEA had larger AUCs, comparing with the AUCs of serum CA 125 and ascites CA 125 (Table 4, Figure 2). Furthermore, ascites CI displayed the best sensitivity, and serum CI displayed the best specificity, when the cut-offs were set to 2919.30 and 2303.13, respectively (Table 4).

**Table 4:** Differentiating PC from TBP by CA 125, CEA and CI

	N	AUC	Cut-off	Sensitivity	Specificity	YI	PV+	PV-
Serum CA 125 (U/ml)	273	0.536 (0.469-0.604)	857.30	0.300	0.914	0.214	87.1%	59.7%
Serum CEA (ng/ml)	251	0.927 (0.891-0.962)	3.64	0.887	0.978	0.865	98.6%	83.3%
Serum CI (U/ml × ng/ml)	244	0.914 (0.876-0.951)	2303.13	0.832	0.989	0.821	99.2%	77.2%
Ascites CA 125 (U/ml)	122	0.753 (0.670-0.837)	1375.50	0.548	0.878	0.425	87.0%	56.6%
Ascites CEA (ng/ml)	95	0.907 (0.848-0.967)	2.43	0.860	0.947	0.807	96.1%	81.8%
Ascites CI (U/ml × ng/ml)	92	0.946 (0.902-0.991)	2919.30	0.907	0.921	0.828	94.2%	87.5%

CI = CA 125 × CEA

Data of 95% CI of AUC are provided in the brackets.

Abbreviations: AUC, area under curve; CA 125, carbohydrate antigen 125; CEA, carcinoembryonic antigen; CI, composite index; N, number; TBP, tuberculous peritonitis; PC, peritonitis carcinomatosa; PV_+_: positive predict value; PV-: negative predict value; YI, Youden index.

## DISCUSSION

CA 125 is found on both healthy and malignant mesothelial and non-mesothelial cells [22] and it was investigated to increase in patients with TBP (See in Supplementary Table 1) [12–20]. However, the sample sizes of these studies were not large, most of which included 20-30 cases, and the main primary locations of carcinoma were ovary and stomach. In addition, nearly half of these studies were case series without control.

Comparing with these studies, our retrospective study included more cases and covered more tumor types. Although the age of patients impacted ascites CA 125, this study did suggest that different diseases affect the extent of elevation of serum and ascites CA 125. Detailedly, CA 125 in TBP group and non-OCA group reached to the level of several hundreds to a thousand. On the contrast, CA 125 in OCA group could reach to the level of several thousands. This distinction might be attributed to different nature of secretory cells in different diseases. As CA 125 is relatively specific in ovarian carcinoma and both tumor and tuberculosis can stimulate the release of CA 125, ovarian carcinoma exerts more potent stimulus on CA 125 secretion than non-ovarian carcinoma and tuberculosis, which might explain the aforementioned phenomenon.

This study also implied the elevation extent of CA 125 was not related with the amount of ascites, differing from the study of E. Zuckerman et al [23]. The discrepancy might be attributed to two reasons. Firstly, ascites due to liver cirrhosis is transudative, but TBP and PC give rise to exudative ascites. The different nature of ascites does not allow the theory proven in liver cirrhosis to be feasible in other populations. Secondly, the accurate method to measure the quantity of ascites has not been fully developed. The prevailing evaluation is qualitative, which is not accurate enough for analysis.

Although OCA-related PC can be distinguished from TBP by different elevation extent of CA 125, differentiation of TBP from non-OCA-related PC still remains a tough problem, because in this study CA 125 levels in TBP and non-OCA-related PC were found to overlap. However, CEA level in non-OCA group (non-OCA-related PC) reached to hundreds, but the CEA levels in TBP group and OCA group (OCA-related PC) were less than ten, which indicated that CEA elevation is less influenced by ovarian carcinoma, followed by tuberculosis.

Although it was supposed that the combined application of CA 125 and CEA was prone to distinguish PC from TBP with a much higher accuracy, comparing with CA 125 alone, both CI and CEA presented the similar diagnostic efficacy in differentiating PC from TBP. Nevertheless, CI still displayed slight diagnostic advantage comparing with CEA, which verified our proposed hypothesis that CI magnified the difference of CEA between TBP and PC by taking CA 125 into account.

Nevertheless, this study still has some disadvantages. Firstly, as a retrospective study, some data were missing, which introduced some bias to the results. Secondly, the epidemiological, geographical and economical factors were less concerned for it was a single-center study. These disadvantages call for sequential prospective studies in future to verify the conclusion drawn from this study.

In conclusion, CA 125 does increase in TBP and non-OCA-related PC, but it elevates more in OCA-related PC. CEA and CI are helpful in distinguishing PC from TBP, and CI is slightly superior to CEA in the differentiation. However, these diagnostic parameters require further validations by prospective cohort studies with larger sample size.

## SUPPLEMENTARY MATERIALS TABLES


