# Supplementary material for: Carbohydrate antigen 125 and carcinoembryonic antigen in the differentiation of tuberculous peritonitis and peritonitis carcinomatosa
Source: Oncotarget. 2017 Apr 21;8(44):78068–75. doi: 10.18632/oncotarget.17355 (PMC5652837; doi:10.18632/oncotarget.17355)
Supplement: Supplementary file 1 [file oncotarget-08-78068-s001.pdf]

Carbohydrate antigen 125 and carcinoembryonic antigen in the differentiation of tuberculous peritonitis and peritonitis carcinomatosa

SUPPLEMENTARY MATERIAL

Supplementary Table 1: Literature review on CA 125 in TBP and malignancies

Author	Research type	Patients	CA 125 results (U/ml)
Bae, 2013 [12]	Retrospective controlled study	TBP: 48 female<br>Ovarian Ca: 370 female	TBP: 418 (range: 25-2702)<br>Ovarian Ca: 1002 (7-42600)
Wang, 2012 [13]	Retrospective controlled study	TBP: 30 female<br>PPC: 38 female	TBP: 344.5 ± 255.0<br>PPC: 2625.5 ± 3923.7
Oge, 2012 [14]	Case series	TBP: 20 female	TBP: 289 ± 186.2
Kaya, 2011 [15]	Prospective controlled study	TBP: 27 (6 male/ 21 female)<br>Ov CA: 24<br>Ga CA: 24 (13 male/ 11 female)	TBP: 229.2 ± 52<br>Ov CA: 2241 ± 565<br>Ga CA: 167 ± 138
Choi, 2010 [16]	Retrospective controlled study	TBP: 17 female<br>PPC: 20 female	TBP: 448 (32-1725)<br>PPC: 1848 (42-14380)
Sharma, 2010 [17]	Case series	TBP: 26 female	TBP: 594.22 ± 770.07
Ulusoy, 2010 [18]	Case series	TBP: 8 female	TBP: 421 (107-725)
Mas, 2000 [19]	Case series	TBP: 6 male and 4 female	TBP: 475.80 ± 106.19
Simsek, 1997 [20]	Prospective controlled study	TBP: 2 male, 9 female<br>Healthy: 7 male, 13 female	TBP: mean 316.6<br>Healthy: 13.8

Abbrevations: Ca, carcinoma; CA 125, carbohydrate antigen 125; Ga CA, gastric carcinoma; Ov CA, ovrain carcinoma; PPC, primary peritoneal carcinoma; TBP, tuberculous peritonitis.

REFERENCES

12. Bae SY, Lee JH, Park JY, Kim DM, Min BH, Rhee PL, Kim JJ. Clinical significance of serum CA-125 in Korean females with ascites. Yonsei Med J. 2013; 54: 1241-7.

13. Wang D, Zhang JJ, Huang HF, Shen K, Cui QC, Xiang Y. Comparison between peritoneal tuberculosis and primary peritoneal carcinoma: a 16-year, single-center experience. Chin Med J (Engl). 2012; 125:3256–60.

14. Oge T, Ozalp SS, Yalcin OT, Kabukcuoglu S, Kebapci M, Arik D, Isikci T. Peritoneal tuberculosis mimicking ovarian cancer. Eur J Obstet Gynecol Reprod Biol. 2012; 162: 105-8.

15. Kaya M, Kaplan MA, Isikdogan A, Celik Y. Differentiation of tuberculous peritonitis from peritonitis carcinomatosa without surgical intervention. Saudi J Gastroenterol. 2011; 17: 312-7.

16. Choi CH, Kim CJ, Lee YY, Kim JS, Song T, Park HS, Kim MK, Kim TJ, Lee JW, Lee JH, Bae DS, Kim BG. Peritoneal tuberculosis: a retrospective review of 20 cases and comparison with primary peritoneal carcinoma. Int J Gynecol Cancer. 2010; 20:798–803.

17. Sharma JB, Jain SK, Pushparaj M, Roy KK, Malhotra N, Zutshi V, Rajaram S. Abdomino-peritoneal tuberculosis masquerading as ovarian cancer: a retrospective study of 26 cases. Arch Gynecol Obstet. 2010; 282:643–48.

18. Ulusoy AN, Karabicak I, Dicle K, Kefeli M, Tosun M, Cetinkaya M, Alper T, Ustun C. Peritoneal tuberculosis in premenopausal patients with elevated serum CA 125. Arch Gynecol Obstet. 2010; 282:639–42.

19. Mas MR, Comert B, Saglamkaya U, Yamanel L, Kuzhan O, Ateskan U, Kocabalkan F. CA-125; a new marker for diagnosis and follow-up of patients with tuberculous peritonitis. Digestive & Liver Disease. 2000; 32: 595-7.

20. Simsek H, Savas MC, Kadayifci A, Tatar G. Elevated serum CA 125 concentration in patients with tuberculous peritonitis: a case-control study. Am J Gastroenterol. 1997; 92:1174–76.
